# Civil and Military Surgery

**Published:** 1861-09

**Authors:** 


### EDITORIAL DEPARTMENT.

### CIVIL AND MILITARY SURGERY.

It is being attempted to separate civil, and military medicine, and surgery by very arbitrary distinctions, to draw very sharp lines of comparison, and virtually make military practice altogether another and almost distinct profession. It is said that a surgeon of high attainment and great experience, is not thereby fitted to discharge readily, and intelligently, the duties of military practice; that, though he may have attained the highest reputation as a surgeon, yet he may never have met an accident peculiar to the field of action; that though he may have been a good family physician in the town where he resided, still he knows nothing of the habits and diseases peculiar to soldiers, does not understand hygine sufficiently to organize his hospital, select his tent ground, provide for ventillation, drainage cleanliness, proper dress, diet, &c. Now it may be well at this time to examine this ground very carefully, since it is intimated that where the surgeons of the regular army, are brought in contact with the surgeons of the volunteer service, the latter should take rank subordinate to the former, on the ground of education and experience in military surgery. It will be admitted that military practice has its peculiarities, and that its details are better understood after experience has made every thing familiar, but we must not admit that the principles of civil surgery are unlike those found to apply in military practice. We cannot appreciate any differences which should disqualify any experienced surgeon, eminently capable of discharging the duties of a civil surgeon, from entering immediately upon the most responsible duties of the military practice. They have never met the accidents peculiar to a field of action; shall we consequently conclude that they have not met cases sufficiently similar to become altogether familiar with the practice which should be adopted? They have not been familiar with the habits and diseases of soldiers: What habits have soldiers, what diseases, with which our most experienced physicians are not familiar? It will be admitted that camp life has its peculiar exposures, and is liable to epidemics influenced by various contingences; yet it will be intimated with great truth that the obscure causes of disease, hidden oftentimes from the civil physician, will also be liable to evade the investigations and experience of the most educated and experienced observer. The same principles of hygine which are perfectly familiar to every well educated, observing, progressive physician, must be ample for guidance in military life. Physicians know far better what *is desirable and proper,* than the circumstances and necessities of the camp, field, and military hospital, will permit to be instituted. They make the best of what they are obliged to endure, not from choice, or ignorance, but from necessity. We venture to say, few surgeons in our volunteer service but are fully competent to appoint all desirable conditions to preserve the soldiers from disease, at least in a degree vastly greater than the circumstances will allow being adopted.

It will be at once conceded that in the regular army we have many practically educated physicians, qualified both by education and experience to render eminently valuable service. We regret that any comparison should be forced upon us, yet we can hardly meet this question without considering the relative conditions of physicians in military, and civil practice. Candidates for admission to the regular army are first graduated in the civil school; after this are subject to a rigid examination, and thus only the well qualified are admitted to military practice; this is done while they are young, and really students in medicine, having as yet no practical knowledge. When once approved as army surgeons, their business is provided or appointed to them, and all the stimulus of business competition is at once withdrawn; they receive their pay, and do the duty assigned them, often acting under the direction of their seniors, and thus failing to cultivate and gain the very desirable attainment of self-reliance. Again, their advancement in position, and increase of salary, is made to depend upon the number of years' service, rather than high professional attainment; their library is necessarily small and incomplete, or if not, they are separated from it; their journals and medical periodicals are few, and irregularly received, or not obtained at all; themselves when advanced to important positions, isolated and excluded from association with professional friends; consultations often impossible, and if not, discouraged, and avoided, upon the preposterous and absurd ground that an army surgeon is capable of taking exclusive charge of any case of disease or accident, however important.

Now, in civil practice, quite the reverse of all this is found to prevail. The young physician when once he has received his diploma has only commenced in the strife for professional success, which must depend mainly, and almost wholly, upon professional attainment; his business, his money, his reputation, *his all* depends upon his own exertions. Years of service only "*advance him backwards*" unless he is ever active and earnest in his efforts for improvement. His aids in this work are usually abundant, and an enlightened public sentiment requires, and demands their acceptance Studious habits, an ample library, and frequent consultations, are required of civil practitioners to gain the confidence and support of the most intelligent and appreciating.

It will be proper to inquire also as to the source of what is really known of the causes of disease and the results of practice even in the military camp and hospital. Who have been most ready to collect, prepare and report the lessons of military practice? The law may have required some statistical reports, which have been made the basis of much valuable information, but certainly military practitioners have contributed sparingly compared with their opportunities for observation and research.

Much that is claimed for the military surgeon will be readily and cheerfully admitted. All that is said of the want of experience in our volunteer surgeons in the time of actual battle, will be also admitted, and the same must be conceded of our military surgeons, since our country has not recently, until now, offered to any this experience.

In concluding this article, we desire only to add—Rank and Subordination are words not proper for Republicans to make free use of. Physicians both of the Volunteer Militia and of the Regular Army, who serve their country faithfully, will have no superiors—will hold *high* and *equal rank.*

